# Polarization and Myelination in Myelinating Glia

**DOI:** 10.5402/2012/769412

**Published:** 2012-12-30

**Authors:** Toshihiro Masaki

**Affiliations:** Department of Medical Science, Teikyo University of Science, 2-2-1 Senju-Sakuragi, Adachi-ku, Tokyo 120-0045, Japan

## Abstract

Myelinating glia, oligodendrocytes in central nervous system and Schwann cells in peripheral nervous system, form myelin sheath, a multilayered membrane system around axons enabling salutatory nerve impulse conduction and maintaining axonal integrity. Myelin sheath is a polarized structure localized in the axonal side and therefore is supposed to be formed based on the preceding polarization of myelinating glia. Thus, myelination process is closely associated with polarization of myelinating glia. However, cell polarization has been less extensively studied in myelinating glia than other cell types such as epithelial cells. The ultimate goal of this paper is to provide insights for the field of myelination research by applying the information obtained in polarity study in other cell types, especially epithelial cells, to cell polarization of myelinating glia. Thus, in this paper, the main aspects of cell polarization study in general are summarized. Then, they will be compared with polarization in oligodendrocytes. Finally, the achievements obtained in polarization study for epithelial cells, oligodendrocytes, and other types of cells will be translated into polarization/myelination process by Schwann cells. Then, based on this model, the perspectives in the study of Schwann cell polarization/myelination will be discussed.

## 1. Introduction

 Cell polarity indicates the presence of structural and molecular asymmetries based on asymmetric distribution of proteins, lipids, and RNAs in cells [1–6]. The cell polarization is ubiquitously important in cellular function, in particular, in multicellular tissues where multiple types of differentiated cells play specific roles. Understanding cell polarization is, therefore, one of the major goals of cell biology. Cell polarization in epithelial cells or other types of cells has been extensively studied. However, cell polarization is a formidably complicated process involving cytoskeletal remodeling, membrane traffic, RNA localization, protein interaction, and intracellular signaling, with feedback to gene expression [7]. In addition, despite the high level of conservation of cell polarity-associated proteins, the interaction between the polarity proteins and other signaling components varies from one cell context to another and from one species to another, which complicates the task of dissecting cell polarization [7]. Thus, the understanding of cell polarization still remains incomplete, and cell polarization is still one of the hottest fields in biology.

On the other hand, cell polarization in myelinating glia, especially in Schwann cells, has been less extensively studied. The myelinating glia, oligodendrocytes (OLGs) in central nervous system (CNS) and Schwann cells in peripheral nervous system (PNS), form the myelin sheath, a multilayered membrane system enabling salutatory nerve impulse conduction and maintaining axonal integrity. The myelin sheath is a polarized structure, and there is some similarity between Schwann cell polarity and epithelial apicobasal polarity [8, 9]. Thus, cell polarization in myelinating glia may be a prerequisite for myelination to start, or progress coordinately with myelination, at least. Studying the mechanism of cell polarization in myelinating glia is therefore critically important in order to elucidate the mechanism of myelination.

The ultimate goal of this paper is to provide insights in the field of myelination by applying the information obtained in polarity study in other types of cells, especially in epithelial cells, to cell polarization of myelinating glia. Thus, in this paper, recent progresses in the main aspects of cell polarity in general are summarized. Then they will be compared with the polarity specific to OLGs. Finally, present status of cell polarization study in Schwann cells is summarized, and then the author will attempt to translate the achievements obtained in polarization study for epithelial cells, OLGs, and other types of cells into Schwann cell biology. Extrinsic polarity cues from extracellular matrix (ECM) focus on dystroglycan function and are separately discussed at the final section. Reviewing all the aspects of cell polarization in general is far beyond the scope of this paper, and the interested readers are referred to other papers [1–6].

## 2. Polarity in Epithelial Cells (or in General)

### 2.1. Basic Themes in Cell Polarization Are Conserved

The commonality of cell polarity reflects a fundamental requirement of localizing different activities to distinct regions of cells. Diverse cell types exhibit diverse polarized phenotypes. However, despite the enormous morphological diversities, fundamental machineries for establishing cellular polarization seem to be largely conserved in the metazoa [5, 10]. The fundamental themes in cell polarization can be typically observed in the process of apicobasal polarization in epithelial cells. First, the cells receive extrinsic cues from adhesion receptors regulating cell-cell contact and receptors for ECM. Second, these cues induce the localization of three major polarity complexes that are highly evolutionally conserved: the Par polarity complex, Crumbs complex, and Scribble complex. These complexes seem to provide crucial intrinsic membrane domain orientational cues directing the formation of distinct cortical domains such as apical, lateral, and basal. Third, these polarity complexes direct the asymmetric localization of proteins in apical and basolateral membranes by regulating polarized trafficking machinery. This machinery sorts proteins by recognizing intrinsic protein-sorting codes via cytoplasmic adaptor complexes present in intracellular membrane compartments and then transports specific proteins to the corresponding specific plasma membrane domains, apical, or basolateral. This machinery is present mainly in membrane compartments including endoplasmic reticulum (ER), Golgi, and endosomes. All eukaryotic cells share common cellular machineries for posttranslational protein trafficking present in ER, Golgi, endosomes, and plasma membrane [11], supporting the idea that diverse cell polarities are based on the common themes. Here we summarize the basic cellular machinery for cell polarization.

### 2.2. Polarity in Epithelial Cells

Epithelial cells are densely packed and form continuous sheets, protecting inner tissues against the external environment, and also serve various functions such as secretion, absorption, and transport. To fulfill these functions, epithelial cells have evolved characteristic apical and basolateral membrane domains. The basolateral membrane contacts adjacent cells and the underlying tissue, whereas the apical membrane faces the lumen of an internal organ. Typically, the apical domain is formed by the brush border of microvilli, which is underlain by a terminal web of actin and spectrin filaments [3]. These are linked to the plasma membrane by the ERM family proteins: Ezrin, Moesin, and Radixin. Since the apical membrane is exposed to hostile environments such as high osmotic pressure or the presence of digestive enzymes, it needs to be particularly sturdy. That seems the reason why the apical membrane is strongly enriched in sphingolipids [12], which, together with cholesterol, have the propensity to form tightly packed membrane microdomains called lipid rafts. The high proportion of sphingolipids and cholesterol in the apical membrane could make it a continuous raft membrane in which nonraft domains are embedded [13, 14]. The basolateral domain is subdivided into a lateral domain that mediates the adhesion between adjacent cells and provides mechanical support for the epithelium and a basal domain that contacts the subcellular tissues. The two membrane domains are separated by tight junctions, which help to prevent mixing of apical and basolateral membrane components and seal the epithelium [15]. The tight junctions contain a number of homophilic adhesion molecules, such as Occludin, Junctional adhesion molecules (JAMs), and the Claudins [3]. These proteins are all clustered by the MAGUK (membrane-associated guanylate kinase-like homology) proteins, ZO-1, and ZO-2, which bind to the cytoplasmic tails of Claudin and Occludin through their N-terminal PDZ domains. Adherens junctions are located just below the tight junctions, providing the main mechanical link between cells. Adherens junctions are characterized by the presence of cadherins and their cytoplasmic adaptor proteins, beta-catenin, and alpha-catenin, which mediate homophilic adhesion with adjacent cells [16]. Another class of homophilic adhesion molecules, the nectins, also localizes to these junctions and links these junctions to the actin cytoskeleton through the adaptor protein, Afadin/Af-6 [16, 17]. The basal domain contacts the ECM or basement membrane, which is enriched in ECM receptors, such as dystroglycan and integrins.

It is worth noting here that, near the apical surface, there is a compartment which is variously termed the subapical compartment (SAC), the apical recycling endosome (ARE), common endosome (CE), or the common recycling endosome (CRE) [18]. This compartment seems to ubiquitously exist in the polarized epithelial cells and is morphologically typified by a branching tubulovesicular network that is clustered in the apical region and extends to the cell periphery. ARE and CE may be distinguished in SAC, as CE recycle cargo from both apical and basolateral surfaces through apical early endosome (AEE) or basolateral early endosome (BEE), whereas ARE seems to be the most distal subapical station relaying apical cargo from CE to the apical membrane [18]. ARE is also biochemically distinguished as a compartment to which Rab11 predominantly localizes.

### 2.3. Three Protein Complexes Controlling Protein Sorting and Trafficking

Three classes of protein complexes have been identified [19–21]: coatomer protein complex-II (COPII) is involved in transport between the Golgi, the plasma membrane, and endosomes. The COPII coat machinery includes the small GTPase Sar1, its transmembrane GEF (guanidine exchange factor) Sec12, and two protein complexes, Sec23/24 and Sec13/31 complexes [22]. COPI complexes are involved in Golgi-ER trafficking and intra-Golgi transport. The COPI coat comprised seven subunits that assemble on the Golgi membrane, generating COPI-coated vesicles [23]. The adaptor protein-clathrin complex (the AP-clathrin complex) is involved in transport between the Golgi, the plasma membrane, and endosomes [5]. Each complex recognizes and selectively recruits target proteins into transport vesicles (nonrecognized proteins are excluded from vesicles), whereas each complex also deforms and sculpts the membrane to produce the membrane vesicle or a tubule. These generic mechanisms are the core machineries of trafficking in all cells and are modified in polarized cells to sort proteins into separate plasma membrane domains.

### 2.4. AP Complexes

AP complex family regulates not only protein sorting associated with endocytosis and exocytosis in the Golgi complex, the plasma membrane, and endosomes, but also the assembly of clathrin scaffolds, which sculpt the membrane to vesicles. Four AP complexes (AP-1~4) have been identified [5]. Each AP complex is composed of two large subunits (alpha, gamma, epsilon, delta, or beta1-beta4; ~100 kDa each), one small subunit (sigma1–sigma4; ~20 kDa), and one medium subunit (*μ*1–*μ*4; ~50 kDa). Together with other proteins such as GGA, AP180, epsin-1, epsin-2, EPS15, beta-arrestin, and ARH that interact with clathrin, the AP subunits recognize and bind specific amino-acid motifs in the cytoplasmic domain of membrane proteins and cluster these proteins into patches on the membrane by assembling a clathrin cage [24, 25]. AP-2 mediates endocytosis from the plasma membrane, whereas AP-1, AP-3, and AP-4 mediate sorting at the trans-Golgi network (TGN) and endosomes. One of the best studied interactions is between the AP-2-clathrin complex and the transferrin receptor (TfnR), which occurs on the plasma membrane. The *μ* subunit of AP-2 (*μ*2) recognizes a degenerate tetrapeptide cytoplasmic domain sorting motif (YxxΦ, in which Φ represent any hydrophobic amino-acid residue), resulting in the clustering of TfnR into clathrin-coated pits on the plasma membrane [26]. These coated pits then bud into the cytoplasm and are delivered to early endosomes, where the sorting motifs including YxxΦ are recognized by *μ*1A and *μ*1B subunits of AP-1A and AP-1B, respectively, in endosomes [27]. 

### 2.5. Basolateral Sorting and Role of AP Complex

Basolateral sorting is directed by Tyr-based motif (YxxΦ) or dileucine-based motif in the cytoplasmic domain [5, 28]. Other basolateral signals are constituted by single leucine/acidic patch motifs as in CD147 [29] or by sequences not yet resembling any other basolateral signal, for example, pIgR [30], NCAM [31], EGFR [32], ErbB2 [33], and TGF-alpha [34].

For example, LDLR has two Tyr-based motifs—a membrane-proximal motif, NPXY, and a C-terminal motif, YxxΦ—in its cytoplasmic domain, and these are required for post-Golgi delivery and the maintenance of LDLR to the basolateral domains [35, 36]. The membrane-proximal motif, NPXY, is important for LDLR endocytosis, and the C-terminal motif, YxxΦ, encodes a signal for sorting to the basolateral domains. The AP complexes that are associated with basolateral protein trafficking are either a ubiquitous AP-1A expressing *μ*1A subunit or an epithelial specific variant of the clathrin-associated AP-1 complex designated as AP-1B, in which the ubiquitously expressed *μ*1A subunit is replaced by a closely related *μ*1B subunit [37, 38]. Association of AP-1B with Tyr-based signals was demonstrated as the absence of *μ*1B leads to a nonpolarized or apically localized protein, a similar effect to that of eliminating the sorting motif. However, diLeu-based signals associated with basolateral polarity are AP-1B-independent [37, 38]. ADP-ribosylation factor 6 (ARF6) is suggested to play a role in AP-1B-dependent sorting from recycling endosomes to the basolateral membrane [39]. Recently, double-knockdown study of AP-1A and AP-1B showed that AP-1A also plays a role in basolateral sorting [27]. Since AP-1A localizes predominantly to the TGN and/or early endosomes, and AP-1B to recycling endosomes this suggests complementary roles of AP-1A and AP-1B in basolateral sorting. AP-4 is also suggested to have a role in basolateral targeting, whereas its signal specificity remains unclear [40]. In addition, another adaptor protein, ankyrins may be associated with E-cadherin trafficking from the Golgi to the basolateral membrane [41].

Recently, clathrin was shown to be required for basolateral plasma membrane protein sorting [42]. Knockdown of clathrin heavy chain in MDCL cells caused loss of basolateral polarity due to a specific defect in the transport and sorting. The affected proteins covered a broad range of basolateral signals, including TfR (tyrosine-independent signal), VSVG (tyrosine signal), E-Cadherin (dileucine motif), NCAM (tyrosine-independent signal), and CD147 (single leucine plus acidic cluster signal) [28].

### 2.6. Apical Protein Sorting and Transport

Rab GTPases play crucial roles in defining apical trafficking routes. Rab4 and 5 are established regulators of early endosomal trafficking. Rab5 is required for the fusion between endocytic vesicles and early endosomes, whereas Rab4 is involved in directing vesicular transport to the recycling endosome [43]. Rab11a is present on the apical recycling endosomes (ARE), where it interacts with myosin-Vb to modulate protein export to the apical domain [44, 45]. Rab8 and Rab10 have been proposed to participate in basolateral targeting from the common recycling endosomes (CRE) and therefore might be involved in the initial stages of the transcytic route to the apical surface in MDCK cells [46, 47]. However, in intestinal cells, Rab8 has been found to regulate apical protein localization [48] and indeed Rab8 also interacts with myosin-Vb [45]. Also there is evidence that Rab13 and Rab14 are involved in apical trafficking [49, 50].

 In apical trafficking, fusion of the transport vesicles with the plasma membrane is mediated by specific vesicle-soluble NSF attachment protein (SNAP) receptors (V-SNAREs) or vesicle-associated membrane proteins (VAMPs) in the transport vesicles and target (t)-SNAREs in the plasma membrane [51]. VAMP7 and VAMP8 seem to differentially regulate membrane fusion of apically destined vesicles that originate, respectively, from the vectorial and transcytotic pathways [52].

Apical sorting motifs are localized in the extracellular, transmembrane, or cytoplasmic domain of proteins [53]. Extracellular domain motifs contain N- and O-linked oligosaccharide chains such as those found in p75 [54] and sucrose isomaltase [55]. The membrane-associated signal can be the transmembrane domain itself as occurs in some viral glycol-proteins, such as haemagglutinin and neuraminidase, but the best described signal is the glycosyl phosphatidylinositol (GPI) lipid anchor [56]. GPI-anchored proteins are sorted into the apical pathway in the Golgi complex. This occurs by GPI-anchored protein oligomerization in lipid rafts [57], which are enriched in glycosphingolipids, sphingomyelin, and cholesterol [56]. Finally, rhodopsin has the cytoplasmic signal and dynein binding sites [43]. 

Now apical transport in epithelial cells is suggested to be frequently mediated by endosomes. Endosomal compartment is comprised of distinct populations of basolateral early endosomes/basolateral sorting endosomes (BEEs/BSEs) and apical early endosomes/apical sorting endosomes (AEEs/ASEs), both of which receive internalized proteins by endocytosis from corresponding membrane domain. Common recycling endosomes (CREs) or apical recycling endosomes (AREs) play a role in recycling of basolateral proteins or apical proteins, respectively. At least, three transport routes mediated by endosomal compartment are suggested. First, GPI-anchored proteins, that are thought to associate with lipid rafts, may be transported to apical domain via AEEs/ASEs associated with Rab4/Rab8/Rab11 [58, 59]. Second, apical proteins reach apical membrane via AREs. This route is raft independent but glycosylation dependent, such as endolyn [58]. Third, the transcytotic route is used by many apical proteins. For example, pIgR is believed to be targeted from the TGN to the basolateral membrane directly or via the BEE/BSE and then traverse the CRE and ARE before arriving at the apical surface [60].

Basolateral (and somatodendritic) sorting signals are often dominant over apical sorting signals. Transcytosis of membrane proteins from the basolateral to apical domains can occur if the basolateral signal is inactivated after newly synthesized proteins are included into basolaterally directed transport vesicles. For example, the adhesion-signaling protein neuronal-glial- (Ng-) CAM has a Tyr-containing, AP-1B-dependent basolateral targeting signal that is inactivated by phosphorylation on reaching the basolateral surface. In the absence of a functional basolateral signal, Ng-CAM is not captured by AP-1B in endosome after internalization and is instead transcytosed to the apical membrane [61]. However, there are also examples of recessive basolateral determinants [62, 63], indicating that the hierarchy of sorting determinants is more complicated or that their relative strength depends on the protein in which they are present.

### 2.7. Role of Lipid Raft 

Lipid raft is a small lipid-rich cluster consisting of sphingolipids (including both sphingomyelin and glycosphingolipids) and cholesterol. It is a membrane microdomain distributed in a sea of phospholipids [64]. Lipid rafts are believed to play diverse roles in cellular functions such as intra- and intercellular signaling, cellular adhesion, cell entry, and molecular sorting and trafficking. On the basis of previous observations that showed that cholesterol and glycosphingolipids were enriched in the apical domain, it was proposed that lipid rafts could act as primary sorters of apical proteins at the Golgi complex [65]. Since all endogenous GPI-anchored proteins expressed by polarized MDCK cells are apically localized [66] and GPI-anchored proteins are incorporated into detergent-resistant membrane domains as they reached the Golgi complex before reaching cell surface [67], GPI-anchored proteins are thought to be transported via lipid rafts. However, whether this change of lipid environment is required for accurate targeting is unclear. In these experiments, rafts were defined as detergent-resistant membranes (DRMs), that is, membranes that resist solubilization with mild detergents such as Triton X-100. Also, the detergent insolubility method was used to identify raft proteins [64]. However, these are rather crude criteria to determine lipid raft as well as raft proteins [53, 64]. Thus, a lack of suitable methods has hampered to get conclusive evidence about raft association [53]. Nevertheless, DRM fractionation remains a valuable tool in the identification of potential raft constituents [64].

 Amino acid sequence CRAC (cholesterol recognition/interaction amino acid consensus domain) may mediate interactions between proteins and lipid rafts. CRAC domain, mediating interactions between proteins and cholesterol, is generally located in the transmembrane domain and is defined as a sequence pattern of L/V-(X1-5)-Y-(X1-5)-R/K [68]. Raft proteins, caveolin and flotillin [69], have CRAC domains [70]. In addition, roles of galectin-9, sorting nexin 18 or FAPP2 in lipid raft clustering, and transport carrier generation associated with apical transport have been suggested [71–74]. 

### 2.8. Controlling Vesicle Fusion at Plasma Membrane Domains

Rab family of small GTPases seem to control many stages of vesicle docking and fusion, especially by having a role in tethering vesicles to their target membranes [75]. At the plasma membrane, the exocyst complex seems to regulate the docking of a subset of vesicles, including basolateral vesicles in epithelial cells [76, 77]. The exocyst is a large complex of at least six proteins, some of which are localized on the target plasma membrane and others are localized to the transport vesicles, along with a Rab family GTPase, which helps to control tethering-complex assembly of an exocyst holocomplex. Another type of vesicle-tethering complex comprises annexins and is present on other plasma membranes, such as the apical membrane of epithelial cells. Annexins bind to membranes in a phosphatidylinositol-3,4-bisphosphage- (PtdIns(3,4)P2-) dependent and Ca2+-dependent manners and self-aggregate. As with components of the exocyst complex, annexins might be present on both vesicles and target membranes [78]. The delivery of some apical proteins in epithelial cells (e.g., the delivery of sucrose-isomaltase [79]) requires annexin II.

Fusion of vesicles with a target plasma membrane is mediated by the SNARE complex, which comprises vesicle (v)-SNAREs such as vesicle-associated membrane protein (VAMP) and target (t)-SNAREs such as syntaxins [80]. In polarized epithelial cells, apical and basolateral vesicles contain different v-SNAREs such as tetanus-insensitive (Ti)-VAMP and VAMP8, respectively [52], and different t-SNAREs are localized to the apical (syntaxin-3) and basolateral (syntaxin-4) plasma membranes [81, 82]. Loss of function of SNAREs leads to a concomitant disruption in the delivery of apical or basolateral vesicles to the plasma membrane [52, 83–85].

### 2.9. Intrinsic Membrane Domain Orientational Cues: Three Complexes Regulating Polarity—PAR Complex, Crumbs, and Scribble Complex

Intracellular complexes, partitioning defective (PAR) and Crumbs and Scribble complexes, are predominantly localized at the cell cortex, providing fundamental orientation cues to identify different regions of the cell cortex.

#### 2.9.1. PAR Complex

The par genes were identified as maternal-effect genes, which are required for cytoplasmic localization in early Caenorhabditis elegans embryos [86]. Seven genes were identified, and they are all necessary for the first asymmetric cell division of the zygote. PAR1 and PAR4 (LKB1) are serine/threonine kinases [87, 88]. PAR2 is a RING-finger domain protein that may function as an E3 ubiquitin ligase [89]. PAR3 and PAR6 are PDZ-domain proteins, which have scaffolding or adaptor functions [90, 91]. PAR5 is a 14-3-3 protein that binds to phosphorylated serine and threonine residues [92]. PKC-3 is an atypical protein kinase C (aPKC). Except PAR2, all of the Par proteins and aPKC are conserved throughout the Metazoa [7].

Polarization in single cell and in multicellular organisms can be induced by a pathway that involves tumor suppressor LKB1 (the mammalian orthologue of PAR4). Activation of LKB1 in epithelial cells results in apico-basal polarization of single cells, even in the absence of cellular adhesion [93], suggesting that LKB1 plays a central role in signaling processes inducing the cell polarity. LKB1 is a master kinase that phosphorylates the activities of at least thirteen downstream effector kinases [94]. One of these effectors is AMP-activated protein kinase (AMPK) family, which have also multiple roles in cells, including regulation of energy production [95]. By being a glucose or energy sensor, LKB1/AMPK pathway is a molecular link between polarity and the metabolic status of a cell. In addition, this pathway seems to play multiple roles in cell, including formation of tight junction and E-cadherin adhesion complexes as well as cell growth via mTOR pathway [4]. Thus, inhibition of this pathway may lead to carcinogenesis [4]. Interestingly, as described in detail later, dystroglycan seems to regulate apico-basal polarity via regulating LKB1/AMPK pathway [96]. AMPK members have several function homologues: the kinase synapses of amphids defective (SAD), the Ser/Thr kinase PAR1, and the family of ELKL-motif kinases (EMKs; also known as microtubule affinity-regulating kinases (MARKs)), which destabilize microtubules [97]. These functional homologues have important roles in the orientation of polarity in neurons and epithelial cells [95].

 Overexpression of PAR1b (EMK1, MARK2) in MDCK cells results in the partial reorientation of the apical membrane to intercellular lumens on the lateral membrane domain, similar to the orientation of the apical (bile canalicular) membrane in hepatocytes [98]. MDCK apical plasma membrane reorientation is accompanied by a change in microtubule organization, such that the minus ends of microtubules localize to the intercellular lumens instead of to the top of the cells. In addition, trafficking of post-Golgi transport vesicles to the apical surface is redirected into an indirect pathway (vesicles first appear at the basolateral domain and then transcytose to the apical domain), as also occurs in hepatocytes [98]. PAR1 homologues in budding yeasts, Kin1 and Kin2, interact with the machinery for vesicle tethering (the Rab proteins and the exocyst complex) and membrane fusion (the SNAREs) [99]. Altogether, these results suggested that PAR1 plays roles in multiple key machineries generating cell polarity.

PAR3 and PAR6, which also bind active Cdc42 [100] and atypical protein kinase C (aPKC), form a complex that is localized to the apical junctional complex in polarized epithelial cells [101–104]. PAR3-PAR6-aPKC seems to play a central role for cell polarization ubiquitously including epithelial cells, as described below in detail [102, 105]. 

#### 2.9.2. Crumbs Complex

 The Crumbs complex comprises transmembrane proteins, Crumbs (Crb1-3 in vertebrates), the PDZ-domain-containing proteins Stardust (PALS1/MPP5 in vertebrates), PATJ (PALS1-associated tight junction protein), and Lin7 [106–108]. Crumbs regulates the identity of the apical membrane [105] and localizes to the apical side of the junctional complex in polarized epithelial cells. Loss of function of either the Crumbs complexes results in defects in epithelial polarity that are due to the reduction of the surface area of the apical plasma membrane domains [105].

#### 2.9.3. Scribble Complex

The Scribble complex comprises Scribble, lethal giant larvae (LGL), and discs large (DLG), regulates the identity of the basolateral membrane, and is localized below the apical junctional complex and along the membrane at cell-cell contacts (lateral membrane in epithelial cells) [102, 105]. Loss of any of these proteins induces loss of cell polarity and usually overproliferates [109].

### 2.10. Complex Interactions between the Three Polarity Complexes and Small GTPase, Especially via aPKC Kinase Activity

The three polarity complexes, Par, Crumbs, and Scribble, regulate cell polarity through complex interactions between them. It is worth noting that the localizations of these protein complexes depend on not only anchoring to membrane proteins or phospholipids but also active exclusion mechanism between each other as described as follows.

First, PAR6 and aPKC colocalize with Crb, Stardust/PALS1, and PATJ, and there is increasing evidence that PAR6 and aPKC are key components of this apical complex. PAR6 interacts directly with PALS1, PATJ, and the C-terminal ERLI motif of Crb3, and both PAR6 and aPKC coprecipitate with components of the Crb complex in mammals and *Drosophila* [107]. aPKC can phosphorylate two conserved threonine residues in the cytoplasmic tail of Crumbs in vitro, which are essential for Crumbs activity in vivo [110]. Genetic analysis showed that aPKC is required in early D. melanogaster development to maintain the presence of the Crumbs complex at the apical membrane, probably by direct phosphorylation [110] or by Crumbs binding, through its PDZ-interaction domain, to PAR6 [111]. Later in development, Crumbs is required to stabilize the PAR3-PAR6-aPKC complex at the apical junctional complex [102, 105]. aPKC has recently been shown to regulate Ezrin at the apical side of polarized human intestinal cells in culture, facilitating the formation of the apical cytoskeleton [112]. Thus, the activity of aPKC in association with the Crumbs complex seems to be a key determinant of apical identity, both through the recruitment and activation of downstream apical complexes and through the inhibition of basolateral determinants. The apical Crumb/PAR6/aPKC complex is regulated by the binding of active Cdc42 to PAR-6. The binding of Cdc42 to PAR-6 induces GTP-dependent switch in PAR-6 conformation via a partial CRIB domain [113], relieving the inhibition of aPKC activity by PAR-6 [114]. Actually, Crumbs, PAR-6, and aPKC delocalize when Cdc42 activity is reduced, leading to defect in actin organization, endocytosis, and adherens junction remodeling [115–118]. The function of Crumbs maintaining apical membrane domain depends on two conserved domains in the cytoplasmic tail of Crumbs, a membrane proximal FERM-binding domain, and a C-terminal ERLI motif [119, 120]. The FERM-binding domain recruits beta_H_-spectrin to the apical side of the cell in *Drosophila*. Also there is ample evidence that the Crumbs complex plays a role in tight junction formation at the border between apical and basolateral domains through recruiting multiple tight junction proteins such as ZO-3, Claudin1, and Angiomotin, which forms a complex with the Cdc42-GAP and RICH1 [121–124]. 

Second, PAR3 localizes slightly basal to the Crumbs complex, where it positions to establish the boundary between the apical and lateral domains. aPKC can also phosphorylate PAR3, decreasing the affinity of PAR3 for aPKC, suggesting that the association of PAR3 with PAR6-aPKC is dynamic [125]. In mammalian cells, PAR3 binds directly to the cell-cell adhesion proteins JAMA [126] and nectin [127], both of which colocalize with E-cadherin at the apical junctional complex. The *Drosophila* PAR3 ortholog Bazooka plays a similar role in the formation of the apical zonula adherens through interaction with Armadillo, *Drosophila* Nectin-like protein, Echinoid, Cadherin, PTEN, and Bitesize [128–130], which in turn recruits ERM protein, Moesin, directing the formation of continuous belt of actin around the apical cell cortex [131]. PALS1 is also required for the trafficking of E-cadherin to the plasma membrane and thereby the localization of the exocyst complex to the plasma membrane [132], indicating that complex feedback mechanisms are involved in the organization of these protein complexes at sites of cell-cell adhesion. Disruption of the exocyst complex subunit, EXO84, results in the loss of Crumbs from the epithelial surface of early D. *melanogaster* embryos [133], suggesting that the exocyst complex has a role in regulating the polarized organization of proteins at the apical junctional complex. The function of PAR3 also depends on Rac exchange factor, TIAM1 [134]. It is via binding of the third PDZ domain and the C-terminal region of PAR3 to the TIAM. Then PAR3 sequesters Tiam1 to prevent inappropriate activation of Rac. PAR3 seems to regulate locally the actin cytoskeleton through Rac or association with LIM kinase2 [135, 136]. Both the Crumbs and Scribble complexes are required to position Bazooka (PAR3) and the adherens junctions between them.

 Third, a member of the Scribble complex, LGL, is also a target for aPKC phosphorylation, and LGL phosphorylation inhibits its localization to the apical cortex [137]. The localization of Scribble proteins also depends on Cdc42, Rac1, cadherin, and actin [138]. Scribble complex may regulate Rac1 via interaction with betaPIX and ADP ribosylation factor GTPase-activating protein GIT1 [139]. Studies in *Drosophila* indicate that mutual antagonism between the apical Crumbs complex and the lateral Scribble complex plays a central role in defining distinct apical and lateral domains in epithelial cells [102, 105]. Overexpression of Crumbs mimics the loss of function phenotype of mutation in *scribble* group genes, and *crumbs* and *stardust* null mutants are partially rescued by reducing the levels of any Scribble complex component.

 In addition, aPKC phosphorylates PAR1, causing PAR1 to bind to PAR5, resulting in the inhibition of the association of PAR1 with the cortex at the apical membrane [140]. On the other hand, PAR1 phosphorylates PAR3 [141] and destabilizes membrane association of PAR3. Thus, the two kinases, aPKC and PAR1, exclude each other from their respective regions of the cell cortex, and it seems that one of the mechanisms is that PAR3/PAR6/aPKC complex is often localized in a complementary pattern to that of PAR1 [7]. Altogether, aPKC seems to be a key kinase which phosphorylates proteins including members of all of the three polarity complexes, interacts with small GTPase signaling, and triggers downstream polarity signaling.

### 2.11. Association of Polarity Proteins with Wnt Signaling

Planar cell polarity (PCP) is an asymmetry at the tissue level rather than at the cellular level. Wnt signaling has been implicated in the PCP [7]. PCP is induced by Wnt ligands activating Frizzled receptor family [142]. Then, adaptor protein, Dishevelled (Dvl), recruits a GEF to activate RhoA [143], which activates the kinase ROCK. ROCK phosphorylates PAR3, in addition to myosin light chain. Dvl is itself phosphorylated by PAR-1 and can be associated with aPKC [144, 145]. The *Drosophila* Frizzled receptor is phosphorylated and inhibited by aPKC, which is recruited to Frizzled through PATJ [146]. Also Scribble and Dlg were shown to bind to components of PCP core machinery including Frizzled receptors and Strabismus [147]. Interaction of Lgl with Dvl has been reported [147].

### 2.12. The Role of Phospholipids in Defining Membrane Domains

 Local asymmetries in the phospholipid content of plasma membrane domains also effect localization of Cdc42 and cell polarity. Local changes in phosphoinositide synthesis are determined by the relative activities of phosphoinositide 3-kinase (PI3K) phosphatase, and tensin homologue (PTEN). PI3K generates phosphatidylinositol-3,4,5-trisphosphate (PtdIns(3,4,5)P3), and PTEN converts PtdIns(3,4,5)P3 to PtdIns(4,5)P2. PI3K activity regulates apico-basal polarization of epithelial cells (Gassama-Diagne et al. 2006) [148]. In polarized epithelial cells, PtdIns(3,4,5)P3 is enriched in the basolateral plasma membrane [148] and recycling endosomes [149], but is absent from the apical in which PtdIns(4,5)P2 is enriched [150]. Both *Drosophila* Bazooka and human PAR3 bind to both PTEN and phosphoinositides [130, 151, 152], and this interaction recruits PTEN to the apical junctions. In addition, PI3K may be recruited to and activated at the lateral membrane by Dlg in response to Cadherin-dependent cell-cell adhesion. Thus, PtdIns(4,5)P2/PtdIns(3,4,5)P3 asymmetry is placed downstream of PAR3 or Scribble complex. However, the strong effects of adding PtdIns(4,5)P2 or PtdIns(3,4,5)P3 to the opposite membrane suggest that this pathway may also feed back to regulate the polarity complexes. For example, Annexin II, a putative scaffolding protein that is required for the delivery of sucrose-isomerase to the apical membrane [79], binds PtdIns(4,5)P2 [153]. Artificially induced accumulation of PtdIns(4,5)P2 on the basolateral membrane causes apical membrane proteins and annexin II to mislocalize to that membrane domain; concomitantly, the former apical membrane is disrupted [150]. Annexin II may in turn recruit other polarity-inducing proteins, such as Cdc42 and aPKC (possibly through Cdc42 as part of the PAR3-PAR6 complex). 

Rush localizes to endosomes via binding to PtdIns(3,4,5)P3. Rush also binds Rab GDP dissociation inhibitor. Rush overexpression study suggested that Rush controls trafficking from early to late endosomes and from late endosomes to lysosomes by modulating the activity of Rab proteins [154]. Recently, a role of phosphatidylinositol 4-phosphate (PtdIns4P) and related proteins, Vps74 and Sac1 in sphingolipid-dependent sorting in Golgi was suggested [155]. In contrast, *μ*2 subunit of AP-2 binds to PtdIns(4,5)P2, and this binding is necessary for endocytosis mediated by AP-2 [156]. Also the mammalian exocyst subunit EXO70 and Sec3 interact with PtdIns(4,5)P2 and PtdIns(3,4,5)P3 [136, 157], suggesting that the polarized distribution of phosphoinositides plays a role in exocytosis. 

## 3. Polarity in Oligodendrocyte

### 3.1. Polarity in Oligodendrocyte: Comparison with Epithelial Cells

During differentiation from oligodendrocyte precursor cells (OPCs), OLGs form a large network of branching processes, and eventually OLGs extend many processes, each of which contacts and repeatedly envelopes a stretch of axon with subsequent condensation of this multispiral membrane forming myelin [158]. Thus OLGs develop quite a unique and complicated cell morphology, and it is not always easy to apply the apico-basal polarity in epithelial cells to these cells. However, still the relationship between the two membrane domains of OLGs, the cell body plasma membrane, and the myelin sheath has some similarity with that between apical domain and basolateral domain in epithelial cells [159]. The myelin-membrane composition differs from that of the plasma membrane. Myelin sheath is a multilayered membrane system that is produced by and extends from OLGs or Schwann cells in the CNS or PNS, respectively. The myelin sheath is highly enriched in lipids. In CNS, cholesterol the glycosphingolipids galactosylceramide (GalCer), and its sulfated analogue sulfatide are main lipid components of the sheath [160]. In addition, several hundreds of proteins have been detected, as revealed by gel-based proteome analysis [161]. The most prominently present are the two major proteins (MBP 30%) and proteolipid protein (PLP 50%). Also the myelin sheath is divided into two parts; compact and noncompact regions that differ in protein composition. In analogy to polarized epithelial cells, the myelin sheath resembles the apical membrane because the myelin sheath is enriched in GPI-anchored proteins, glycosphingolipids (such as GalCer and sulfatide), and cholesterol. On the other hand, the viral model protein HA of influenza virus, a marker for vesicle-mediated transport to apical membrane involving tSNARE syntaxin 3, localizes to the plasma membrane, and VSVG, a basolateral marker involving syntaxin 4, localizes to the myelin sheath. Tyrosine motif dictating basolateral sorting via its association with the AP1B adaptor molecule [26] is also used for myelin-directed targeting. Also in OLGs, transcytosis, in which proteins or lipids to the plasma membrane transported to plasma membrane are transported again to the myelin sheath, seems to operate. Thus the polarity in OLGs is complicated, and the idea that myelin sheath in OLGs corresponds to the apical domain in epithelial cells should be always viewed with some caution.

### 3.2. Myelin Protein Sorting and Transport in OLGs

Most myelin proteins are transmembrane proteins and are thus synthesized at the ER. From there, they are transported by vesicular transport to the Golgi apparatus and further to the plasma membrane. Similarly most myelin lipids are most likely transported by vesicles to myelin membrane. In analogy with epithelial cells, myelin proteins may follow the way apical proteins are sorted and transported. Actually, presence of many GPI-anchored proteins associated with myelin sheath as well as detergent insolubility of several myelin proteins suggests the similarities of myelin proteins to apical proteins. Here, examples of myelin protein transport, MBP, and PLP in OLGs will be reviewed.

#### 3.2.1. MBP Transport

MBP is transported towards the myelin in the form of its mRNA [162]. Thus, MBP mRNA is mainly localized in myelin membrane domain, and locally translated to MBP proteins, forming important part of cell polarity in myelinating OLGs. Actually, RNA localization and local translation are important in cell polarization in several other situations. The mRNAs for Crumbs and Pals1 are enriched near the apical surface in *Drosophila* epithelial cells [163, 164]. One of the reasons of mRNA localization is to regulate translation quickly in response to extracellular signals. In the case of MBP also, mRNA localization in myelin sheath domain may enable timely translation of MBP mRNA into proteins and insertion into myelin membranes during myelination process. In addition, MBP protein is highly basic and, hence, extremely adhesive. By transporting as mRNA, it prevents inappropriate or deleterious adhesive interactions during transport. In addition to MBP, several other proteins such as MOBP, CAII, and tau are also transported as mRNA to the myelin membranes [165, 166].

MBP gene encodes two MBP families, classic MBP and golli-MBP, containing 3 additional exons located upstream of the classic MBP exons. Classic MBP are exclusively expressed in myelin membranes, and consist of different isoforms, mainly distinguished by the inclusion or exclusion of exon 2. The different isoforms of MBP may localize to different microdomains in the myelin membrane according to distinct, isoform-dependent distribution in detergent-resistant microdomains [167], suggesting multifunctionality of this protein in myelin maintenance. 

Targeting of MBP mRNA is a multistep process that involves nuclear transport, assembly into transport particles, trafficking and anchoring to the target site, and finally activation of translation [162]. The first stepis that nuclear export of MBP mRNAs is likely regulated by QKI RNA binding proteins, which bind directly to the 3′ unrelated region (UTR) of MBP mRNA [168, 169]. Interestingly, QKI RNA binding consensus sequences are found in not only MBP but also Krox-20, MAP-1B, and p27KIP1 [168, 170–172]. QKI proteins function in not only MBP mRNA nucleo-cytoplasmic transport but also mRNA stabilization of MBP and p27KIP1. p27KIP1 plays a role in differentiating OLGs and Schwann cells by inducing exit from cell cycle. Thus, QKI proteins seem to have multiple synergistic effects promoting myelination. Recently, it was reported that, in quaking viable (qk(v)/qk(v)) mutant mouse, PLP was downregulated and SIRT2 protein was reduced whereas SIRT2 mRNA was unaffected [173], suggesting that QKI proteins still have further functions on PLP and SIRT2, which remain to be revealed.

 In the cytoplasm, MBP mRNA is incorporated into “granules”, along with protein synthetic machinery such as ribosomal RNA and elongation factor [174]. This incorporation is mediated by the mRNA binding factor hnRNP A2 to hnRNP A2 response elements in RNA [175]. During transport, translation is suppressed by binding of the translational inhibitor hnRNPE1 [176]. 

 Whereas details of the final step, anchoring MBP mRNA granules to the target site and activation of translation, remain unclear, Kif1b is suggested to be required for the localization in the processes of myelinating OLGs [177]. Also the roles of Fyn activation or TOG for the translation have been suggested [178, 179]. ATPase N-ethylmaleimide sensitive factor, a cofactor in SNARE-mediated membrane fusion, and sec8, an exocyst component and a central player in OLG vesicle transport, are required for MBP expression in OLGs, suggesting that mechanisms of MBP mRNA transport may be similar to that of vesicular transport [162].

#### 3.2.2. PLP Transport

 PLP is an integral membrane protein, composed of 276 amino acids and is predominantly expressed in OLGs [162]. The PLP gene is alternatively spliced during development to encode DM20 and PLP. Both proteins consist of four transmembrane domains with one intracellular loop and two extracellular ones. DM20 differs from PLP by a hydrophilic internal 35 amino acid segment deletion in the intracellular loop. PLP plays a major role in the correct apposition of the extracellular leaflets of the membrane, thereby stabilizing the multilayered myelin membranes upon compaction. 

PLP is synthesized in the ER and transported via vesicles to Golgi. Then PLP is transported from the TGN to the plasma membrane of OLG cell body. From there, PLP is internalized and transported to late endosomes/lysosomes by a clathrin-independent but cholesterol-dependent mechanism [180, 181]. Soluble signal from neurons induces transport of PLP from late endosomes to the plasma membrane [181], probably via regulation of Rho GTPase activity [182]. Actually, MAG and PLP are localized in endosomal compartment [181, 183, 184]. PLP is localized in late endosomes/lysosome without colocalization with other myelin proteins such as MAG and MOG [159, 184]. In addition, when stably expressed in HepG2 cells, localization of PLP at the apical membrane is followed by transcytosis to the basolateral membranes [162]. Thus, PLP seems to reach the myelin via a transcytotic pathway independent from other myelin proteins. While the role of sulfatide is suggested in PLP transport [185, 186], it remains uncertain whether PLP can reach the cell surface of OLGs and other nonpolarized cells in absence of sulfatide [187]. Interestingly, in Oli-neu cell line and the human oligodendroglioma-derived cell line HOG, a MAL-2 containing compartment has been identified. MAL-2 is an ARE/SAC protein and is thought to be an essential component of the basolateral-to-apical transcytosis machinery. The MAL-2 containing compartment in OLG cell lines shared main features of ARE/SAC such as colocalization with rab11a, sensitivity to microtubule disruption with nocodazole, and lack of internalized transferring, suggesting that the MAL-2 containing compartment is a structure analogous to the ARE/SAC [188]. In addition, actually, PLP was shown to colocalize with MAL-2 in vesicles and tubulovesicular structures in HOG cell line, supporting the transcytotic model of PLP transport [189]. 

Also, evidence revealing the mechanisms of docking and final insertion of PLP into the myelin sheath is now emerging. OLGs express SNAP-29, a tSNARE containing a binding domain for rab3A in its N-terminus, during myelination [190, 191]. Overexpression of PLP and rab3A in HEK293 cells promoted surface directed trafficking of PLP. Therefore, SNAP29/rab3A system may play a role in PLP trafficking during myelination. In addition, two independent transport pathways of PLP associated with R-SNARE VAMP3 or VAMP7 were suggested in primary OLG [190, 192]. VAMP3 and its acceptor, syntaxin-4, seem to mediate PLP transport to surface membrane via recycling endosome, and VAMP7 and syntaxin-3 seem to mediate it via late endosome/lysosome as part of a transcytosis pathway. 

### 3.3. Membrane Rafts in OLGs

Unusually high concentration of galactosphingolipids (GSLs) and cholesterol in myelin sheath is reminiscent of the apical membranes of epithelial cells [53]. In addition, the myelin sheath maintains a highly specialized region known as the paranode, the portion of each myelin segment that immediately flanks the node of Ranvier [193, 194], and the paranode is highly enriched in putative membrane raft proteins [195–197]. Mice that lack myelin sphingolipids do not form proper paranodes [198–201]. These findings led to the analysis whether lipid rafts, membrane microdomains characterized by the enrichment in GSLs and cholesterol, are involved in the trafficking of myelin proteins or paranodal proteins. 

Incorporation of proteins into rafts depends predominantly on lipid modifications such as palmitoylation or the attachment of a GPI-anchor. Importantly, many myelin proteins have such lipid modifications. For example, contactin and NCAM120 are GPI-linked proteins, whereas the palmitoylation of PLP at three cysteins in the N-terminus is essential for its targeting to the myelin sheath [202]. Association of NF155 with lipid rafts was demonstrated by a combination of in situ extraction with TritonX-100 or hydroxylamine and fluorescent microscopy [64]. After treatment of optic nerve with Triton X-100 (TX100), NF155 was readily extracted at ages when rafts are absent but remained clustered when rafts are present. Also spinal cord was treated with hydroxylamine, which cleaves thioester bonds that covalently attach palmitic acid to cysteine residues in proteins. After treatment with hydroxyl amine, NF clustering was specifically lost. These results suggested that NF155 was associated with lipid raft probably through palmitoylation.

Several noncompact myelin proteins, notably the GPI-linked proteins, contactin and NCAM [203], as well as CNP, MOG [204], and NF155 [195, 205] are at least partially resistant to TX100-extraction. In contrast, another noncompact myelin protein, MAG, is soluble in TX100. MAG is, however, resistant to extraction with lubrol WX [206]. Major compact myelin proteins, PLP and MBP, are soluble in TX100 [180, 207]. However, both proteins are resistant to extraction with CHAPS, whereas GPI-anchored proteins are CHAPS-soluble [167, 180]. Importantly, both cholesterol and glycosphingolipids are required for the CHAPS-insolubility of PLP, and PLP is CHAPS-soluble in cells that do not express GalCer and sulfatide [180]. These results suggest that noncompact myelin proteins, contactin, NCAM, CNP, and MOG; another noncompact myelin protein, MAG; and compact myelin proteins, PLP and MAG, are differentially associated with lipid raft, indicating that the way of association of myelin proteins with lipid raft may play a role in myelin protein sorting. The role of CHAPS-rafts is exemplified by the experiment showing that PLP is recovered from CHAPS-insoluble membrane fraction enriched in myelin lipids [180]. The role of TX100-rafts in the formation of noncompact myelin is exemplified by several findings [159]. NF155 becomes TX100-resistant during OLG differentiation in vivo and in vitro [195, 205]. Both the association of NF155 to TX100 rafts and the localization of NF155 in the paranodal junction are perturbed in ceramide galactosyl transferase (CGT) KO mice, lacking GalCer and sulfatide [195, 208]. Inhibitor of matrix metalloproteases reduces the association of NF155 to TX100-rafts [159] and at the same time impairs the transport of NF155 into the OLG processes [209]. Actually, the establishment of paranodal junction in the optic nerve coincides with the raft association of NF155 [195]. Another myelin protein, proteolipid MAL, may be associated with rafts, because MAL interacts with sulfatide [210]. Interestingly, in epithelial cells, MAL is required for the transport of proteins from the TGN to the apical membrane [211]. In MAL KO mice, myelin sheath is initially normal. However, the levels of several proteins, in particular, MBP, L-MAG, and NF155 are reduced. In addition, raft association and localization of NF155 in the paranodes are perturbed later in life, indicating that MAL may be important for the maintenance of the paranodal junction [212].

 In OLGs, there is an example showing that raft formation forms a platform for intracellular signaling. Activity of the Src family kinase fyn required raft association [213]. The prevention of raft formation strongly inhibits fyn phosphorylation of tau [214]. Raft formation induced by the MAG antibodies increased fyn phosphorylation with a concomitant loss of beta subunit phosphorylation of the heterotrimeric G-protein complex and lactate dehydrogenase and the loss of actin polymerization possibly through a calcium/calmodulin signaling cascade [215]. In contrast, MOG antibodies did not effect signalings by fyn, focal adhesion kinase, or MAP kinase, but the phosphorylation of beta-tubulin and the beta subunit of the G-protein complex were increased [216]. The results suggest that different types of rafts can induce specific signaling cascade in OLG.

 Lipid rafts also seem to play a role in sorting of myelin components [159]. Rafts have been implicated in non-clathrin-mediated endocytosis of OLGs [181, 182]. PLP is internalized from the plasma membrane by cholesterol-dependent and clathrin-independent endocytosis [181]. Also there are several findings suggesting that plasma membrane endocytosis/endosome system plays a role in sphingolipids sorting [159]. Endosomes of polarized HepG2 cells sort different sphingolipids into vesicles, which are destined to the basolateral and apical membrane, respectively [217]. Sphingolipid probes, BODIPY-sulfatide, and BODIPY-lactosylceramide, incorporated into the plasma membrane, are internalized and differentially distributed to endosomes most obviously in mature OLGs [218]. Altogether, these data suggest that the plasma membrane endocytosis/endosome system may play a role in raft-mediated sorting to the myelin sheath. In addition, in mice lacking galactocerebroside and sulfatide, NF155 associates with neither rafts nor clusters in the paranode [194, 198, 208] and paranode stability is lost with age [199], whereas, in mice lacking only sulfatide (CST KO), are capable of clustering NF155 in the paranode suggesting that galactocerebroside is essential for proper NF sorting [201].

## 4. Polarity in Schwann Cells

### 4.1. Anatomical Polarity in Myelinating Schwann Cells

 Schwann cells myelinate a single axon with a relationship of 1 : 1. Axons more than 1 *μ*m in diameter are myelinated. Myelin sheaths comprised of multiple wraps of compacted Schwann cell membrane, is localized inside adjacent to axon, with thin cytoplasm (adaxonal compartment) intervened. The nucleus is located on the outside of the sheath. Most of the cytoplasm is present on the outside of the sheath (abaxonal compartment). The rough ER and Golgi are located in a perinuclear region, and newly synthesized proteins are thought to travel in cytoplasmic channels on the outside of the myelin sheath [219]. The Schmidt-Lanterman clefts, cytoplasmic channels, spiral through the myelin sheath and provide a conduit between the inner and outer cytoplasm. On the surface of Schwann cell outer (abaxonal) membrane, basement membrane is formed. Thus myelin sheath is radially polarized. In the abaxonal membrane, ECM receptors such as dystroglycan and integrin are expressed [220], and the adaxonal membrane is enriched in adhesion molecules that mediate interaction with the axon such as MAG [221].

 Myelinating Schwann cells also exhibit a striking longitudinal polarity comprised of nodes of Ranvier, paranodes, and internodes [8]. Nodes of Ranvier are gaps of about 1 *μ*m between each myelin segment where the axon is exposed to the extracellular environment. The nodes are contacted by hundreds of interdigitating microvilli that project from the end of the Schwann cell to closely appose the nodal axolemma. 

Nodes are flanked on either side by the paranodes. At the paranode, lateral edges of myelin sheath spiral around the axon, forming the axoglial junction [8]. In longitudinal sections, this structure has the appearance of a series of loops, with the outermost loops closest to the node. The paranodal region is the site of closest apposition between the membranes of axon and myelinating Schwann cells. Based on their morphologic similarities to invertebrate septate junctions, the paranodal junctions are referred to as septate-like. In addition, there are several types of junctions between the paranodal loops themselves [222]. Tight junctions seal off the ends of the paranodal loops from the periaxonal space. Adherens junctions between Schwann cell paranodal loops [223] provide structural integrity. Gap junctions facilitate transfer of small-molecular-weight substances between the paranodal loops of Schwann cells. Gap junctions are also present in the SL incisures and are important for peripheral myelin sheath maintenance. NF155, the 155 kDa isoform of neurofascin, is expressed in the paranodes of myelinating glia [224, 225]. NF155 is a likely ligand of Caspr-contactin complex expressed on apposing axolemma.

### 4.2. Roles of Myelin Protein Trafficking in Myelination Revealed by Studies for Demyelinating Charcot-Marie-Tooth Disease

Over the last 15 years, mutations in a variety of genes have been identified causing Charcot-Marie-Tooth disease (CMT) [226]. Owing to studies pursuing to elucidate how these mutations cause myelin defects in CMT, ample evidence accumulated showing that myelin protein is transported by intracellular membrane system: ER, Golgi, and endosome/lysosome system in Schwann cells, as shared by other cell types in polarized protein transport. Also these studies showed that not only myelin protein transport but also endocytosis or endosome recycling plays a role in myelination.

#### 4.2.1. MPZ/p0

MPZ is synthesized in the ER and seems to be sorted in the specific vesicles on exit from the TGN and targeted to the Schwann cell plasma membrane [227]. MPZ-truncating mutation (producing truncated MPZ protein), mutation of serine 204 to alanine or at a nearby presumed PKC substrate motif (198RSTK201) and frameshift mutation Q187PfsX63 cause retention of MPZ in ER, Golgi, or TGN, suggesting that the cytoplasmic domain is important in MPZ trafficking [228–230]. The finding by Xu et al. [231] that calphostin C, a PKC inhibitor, abolished the adhesion of cells expressing MPZ supports the contention that PKC-mediated phosphorylation is necessary for MPZ trafficking. In addition, PKCalpha specifically associates with MPZ. Interestingly, curcumin, which is derived from the curry spice turmeric, is capable of releasing mutated MPZ protein from retention in the ER and abrogates aggregation-induced apoptosis in cell culture [229]. MPZ was shown to be abundantly present in late endosomes/lysosomes. Also downregulation of Rab27a, a small GTPase required for the trafficking of the secretory lysosomes to the plasma membrane, largely blocked lysosomal exocytosis in Schwann cells and reduced the remyelination of regenerated sciatic nerve, suggesting that lysosomal exocytosis is also important in remyelination [232]. Thus MPZ may exit the TGN and reach plasma membrane via Rab27a-positive secretory lysosomes. Actually during the first postnatal week, clathrin-coated pits are prominently associated with rat myelin membranes, indicating that active exocytosis or endocytosis occurs during this maximal period of myelin formation [227]. It is reported that MPZ required cholesterol for exiting ER and reaching myelin compartment. Cholesterol dependency of MPZ trafficking was mediated by CRAC motif [233]. In addition, MPZ, MAL, and PMP22 were recovered along with GPI-anchored protein CD59 from TX100 rafts, suggesting that the transport of these myelin proteins is associated with lipid rafts [234].

#### 4.2.2. PMP22

In severe neuropathy-associated PMP22 mutant forms, these mutated proteins are retained in the ER or in the intermediate component [235–240]. Overexpression of PMP22 induces the accumulation of PMP22 in PtdIns(4,5)P2-positive pool of vacuoles [241]. 

#### 4.2.3. MBP

 Little is known about MBP transport in Schwann cells. However, recently, Larocque et al. suggested that QKI proteins play a role in myelination via multiple functions including MBP and p27KIP mRNA transports [242], suggesting that MBP is also transported as mRNA in Schwann cells. 

#### 4.2.4. MTMR2

Myotubularin-related protein-2 (MTMR2) or MTMR13/SBF2 genes are responsible for the severe CMT disease. Myotubularin-related proteins (MTMRs) constitute a large family of phosphoinositide lipid 3-phosphatasese with specificity for Ptdins3P and PtdIns(3,5)P2 [243]. Interaction of MTMR2 and SBF2 dramatically increases the enzymatic activity. Because Ptdins3P and PtdIns(3,5)P2 are anchor sites on membranes for effector proteins of early and late phases of the endocytic process [244], it is suggested that MTMR2 plays a role in myelination through its function regulating endocytosis.

#### 4.2.5. Dynamin 2

As a large GTPase, Dynamin 2 (DNM2) belongs to a family of proteins that regulate membrane trafficking from TGN and actin-based cytoskeletal dynamics [245, 246]. DNM2 is recruited to cell membranes and critically involved in “pinching” of newly formed clathrin-vesicles using GTP hydrolysis to tighten and deform membranes. Actually, recently, DNM2 CMT-causing mutants were shown to cause not only dysmyelination but also inhibition of clathrin-mediated endocytosis [247]. Also all known CMTDIB-causing mutations are clustered in the DNM2 pleckstrin-homology domain, which mediates interactions with phosphoinositides [248]. The mutated proteins show reduced binding to vesicles and reduction of receptor-mediated endocytosis [249]. Altogether, DNM2 plays a role in myelination through its function regulating protein trafficking, especially mediating endocytosis via its function of “pinching” vesicles. Other genes encoding proteins related to this function such as WASp, Arp2/3, and Cip4 in *Drosophila* may be worth examining in CMT patients [116].

#### 4.2.6. Small Integral Membrane Protein of the Lysosome/Late Endosome (SIMPLE)

The dominant demyelinating neuropathy CMT1C is caused by mutation in SIMPLE [250]. SIMPLE is thought to function in the overall process of lysosomal sorting and the control of protein degradation. SIMPLE interacts with the E3 ubiquitin ligase NEDD4, which plays a role in targeting membrane proteins by mono-ubiquitination for lysosomal degradation [251–253]. SIMPLE binds also via an N-terminal P(S/T)AP motif to the tumor suppressor gene 101 (TSG101), which acts downstream of NEDD4 and sorts ubiquitinated substrates into multivesicular bodies, ultimately leading to degradation in lysosomes [253]. SIMPLE has been found in association with the plasma membrane, the Golgi apparatus, and lysosomes [253–255]. Therefore, SIMPLE may play a role in myelination through its function on protein trafficking, especially through protein degradation in lysosome, while mechanistic details are unclear.

#### 4.2.7. FGD4/Frabin

In the subtype of CMT4H, autosomal recessive demyelinating form of CMT, mutations were identified in FGD4, encoding FRABIN. FRABIN is a GDP/GTP nucleoid exchange factor (GEF) specific to Cdc42 [256, 257]. In these mutations, PH and FVYE domains, involved in interactions with different forms of phosphoinositides, were suggested to be lost.

#### 4.2.8. SH3TC2

Mutations in SH3TC2 are associated with CMT4C. SH3TC2 was identified as a protein regulating endosome recycling through interaction with Rab11 [258, 259], suggesting that endosome recycling plays a role in myelination.

### 4.3. Localization of Polarity Proteins in Schwann Cells

Recently Özçelik et al. reported the localization of polarity proteins in myelinating Schwann cells of mature mouse peripheral nerve [260]. Basolateral markers, Scrib/LGL/DLG1/syntaxin4/sec8/PI3K/PH-AKT (PtdIns(3,4,5)P3 probe), are localized in the abaxonal domain and partially colocalized with E-cadherin of adherens junctions at the outside edge of SL incisures. In paranodal loops, these markers are localized in the outer region of the loops. On nerve cross sections, the basolateral markers colocalized with integrin beta1 in the abaxonal domain. Apico-junctional markers PAR3/aPKC are found in SL incisures, where both PAR3 and aPKC partially colocalized with E-cadherin in adherens junction. PAR3 and aPKC are also expressed in paranodal loops, where they partially colocalized with E-cadherin. Apical markers, PALS1/MUPP1 (PATJ homolog)/annexin A2/PTEN/PH-PLC delta (PtdIns(4,5)P2 probe), are found in SL incisures and the adaxonal domain. In paranodal region, apical markers appear to localize in the inner part of paranodal loops [260, 261]. These data indicate that myelinating Schwann cells are polarized on a radial axis and that this polarity is molecularly similar to the epithelial cell polarity, suggesting common control mechanisms. In analogy to epithelial cells, the abaxonal membrane is a basolateral-like domain, and the incisura-adaxonal domain is an apical-like domain. Interestingly, silencing of PALS1 resulted in a severe reduction of myelin sheath thickness and length. In addition, PALS1 is required for the normal polarized localization of sec8 and syntaxin4 and for the distribution of E-cadherin and myelin protein PMP22 and MAG at the plasma membrane, suggesting that PALS1 is essential in the radial and longitudinal extension of the myelin sheath and likely involved in membrane trafficking of myelin proteins. The localization of apical markers in SL incisures suggests that these structures are apical-like. Yet the outside edge of SL incisures also contains basolateral markers, suggesting that adherens junctions, which are mainly localized on the outside edge of SL incisures [262, 263], form the boundary between abaxonal and incisura-adaxonal domains, just as they form the boundary between apical and basolateral domains in epithelial cells [264]. PMP22 and MPZ are expressed in tight or tight-like junctions of the apical domain of epithelial cells [265–267]. In addition, MBP binds to PtdIns(4,5)P2 [268], which is an apical marker [150]. Altogether, adaxonal membrane domain including the compact myelin and SL incisura may correspond to apical-like domain and abaxonal membrane domain to basolateral-like domain. Considering anatomical and molecular polarity of Schwann cells described previously, Schwann cell microvilli at nodal region may be included in apical domain. Paranodal regions enriched in cell junctions including tight junctions, septate junctions, and zona adherens seem to be similar to lateral membrane domains.

## 5. Extrinsic Membrane Domain Orientational Cues Provided by ECM

Cell adhesion, both to the substratum (ECM) and to other cells, is important in establishing the polarized orientation of cells. Individual epithelial cells that are grown in suspension, in the absence of cell-cell and cell-ECM adhesion, do not develop polarity, but instead undergo programmed cell death (anoikis). When grown on a surface, even single epithelial cells develop an apico-basal axis of polarity. When this surface has biological relevance, such as a surface of laminin-containing ECM, neurons specifically form an axon [269], single mammary epithelial cells selectively secrete beta-casein from the apical surface [270], and 3D epithelial cysts polarize correctly [271]. However, not only adhesion to the ECM but also cadherin-dependent cell-cell adhesion are necessary for MDCK cells to develop authentic apico-basal polarity [272]. In general, cell-substratum adhesion is sufficient to define a noncontacting apical membrane accumulating apical markers, but is not sufficient to localize proteins at the basolateral membrane [3]. Epithelial cell-cell adhesion in suspension culture induces the segregation of basolateral membrane proteins to the cell-cell contacts and induces the trafficking of apical proteins to the free surface [273]. 

### 5.1. Role of Dystroglycan in Epithelial Cell Polarization

As a major component of basement membrane, laminin plays a role in generating and maintaining apico-basal polarity in epithelial cells by providing extracellular cues [274]. As a laminin receptor besides integrins, dystroglycan, a member of dystrophin-associated glycoprotein complex (DGC), is ubiquitously expressed in epithelial cells and is thought to be associated with laminin signaling [275, 276]. Several dystroglycan ligands such as laminin and perlecan are also present in epithelial basement membrane [277]. Inside the cell, the WW domain of dystroglycan binds to dystrophin, which recruits most of the other DGC components, such as syntrophins and dystrobrevin. Dystrophin also binds F-actin. Mammals have two dystrophin paralogs, utrophin and dystrophin-related protein 2 (DRP2), and partial redundancy between these genes was reported [278]. Thus, DGC provides the link between ECM and submembranous cytoskeleton.

There is ample evidence showing association of laminin or dystroglycan with cell polarization. Antibodies against laminin alpha1 were shown to block epithelial polarization in kidney organ culture [279]. Laminin was required for normal localization of PAR3, PAR6, and normal generation of polarity in pharyngeal precursor cells in *C*. *elegans* [280]. Also, the addition of laminin to cysts, which have already polarized, can invert this polarity by binding to integrins [271, 281]. It does this indirectly by activating small GTPase Rac, which in turn is necessary for the deposition of laminin [271, 272]. Interestingly, the reversal of polarity induced by knockdown of Rac or integrins can be rescued by inhibiting RhoA or its downstream effectors, Rho kinase I and Myosin II [282], suggesting that integrin and Rac1 maintain cell polarity at least partly via inhibiting RhoA-ROCK1-Myosin II pathway. The association of small GTPase signaling with dystroglycan is possible because the association of dystroglycan with Rac1 and Cdc42 is reported in some cell lines or other types of cells such as skeletal muscle or oligodendrocyte [283–288]. However, it remains uncertain whether dystroglycan plays a role in the cell polarization of MDCK cells by mediating laminin-induced small GTPase signaling. Interestingly, components of DGC are suggested to interact with polarity protein PAR1. In MDCK cells, PAR1 interacts with DG complex and is required for the formation of a functional DG complex [289]. In addition, the 8th and 9th spectrin-like repeats (R8 and R9) of utrophin, one of the members of DGC, interact with PAR1b, and R9 is specifically phosphorylated by PAR1b [290].

Dystroglycan plays a role in establishment of oocyte polarity in *Drosophila*, and both putative SH3 binding site and WW domain binding sites (binding sites for dystrophin) located at the C-terminal end of dystroglycan are important for this function [291, 292]. In cultured mammary epithelial cells, dystroglycan was required in laminin-induced polarity, beta-casein production, and laminin assembly at the step of laminin binding to the cell surface [293]. In addition, dystroglycan, or perlecan, was shown to be required for apico-basal polarity under energetic stress in *Drosophila* [96], including the polarized localizations of three major polarity complexes, aPKC/PAR6 (apical), Crumbs complex (apical), and Scribble complex (basolateral). Starved dystroglycan or perlecan null cells had a defect in Myosin II activation by AMPK (the low-energy epithelial polarity pathway). Dystroglycan was required both for activation and localization of myosin regulatory light chain in starved follicle cells. It seems likely that binding of perlecan to dystroglycan controls epithelial polarity under low-energy conditions by signaling through the SH3-binding domain. There is also a possibility that localization of dystroglycan-perlecan complex was induced by LKB1 (the mammalian orthologue of PAR4)/AMPK signaling, and this positioning may be a prerequisite for subsequent steps in the polarization cascade [294]. AMPK is indeed required for the restriction of dystroglycan to the basal surface of the basolateral plasma membrane under conditions of energy stress [295].

Interestingly, expression level of alpha-dystroglycan correlated with the ability of breast tumor cell lines to polarize in the presence of basement membrane. Overexpression of alpha-DG enhanced the ability to polarize and reduced their tumorigenic potential in nude mice [296]. Also, in highly metastatic cancer cell lines, alpha-dystroglycan frequently lacked laminin binding by silencing of the like-acetylglucosaminyltransferase (LARGE) gene [297], and exogenous expression of LARGE in these cells restored the normal glycosylation and laminin binding of alpha-dystroglycan, leading to reduced cell migration in vitro. These results suggest that laminin-dystroglycan interaction antagonizes the properties of cancer cells such as proliferation and migration probably via enhancing epithelial cell-type polarity formation. In this context, it is worth noting that epithelial-mesenchymal transition (EMT) generated mesenchymal type-cancer stem cell-like cells from mammary epithelial cells, suggesting that loss of epithelial cell apico-basal polarity may be associated with carcinogenesis. Dystroglycan may antagonize EMT through keeping epithelial cell polarity by providing extrinsic polarity cues. Actually, involvement of dystroglycan in EMT was suggested [298]. Whereas the mechanism of how loss of apico-basal polarity leads to carcinogenesis remains unknown, inhibition of AMPK leads to carcinogenesis. Therefore, LKB1/AMPK may be one of potential candidates linking cell polarity and carcinogenesis. In addition, ΔN isoform of p73, a member of the p53 family implicated in tumor suppression, is suggested to maintain mammary epithelial cell polarity via suppressing EMT [299]. PTEN, a dual-function phosphatase with tumor suppressor function, interacts with E-cadherin, and the interaction plays a role in polarity formation and growth arrest in human mammary epithelial cells [300]. Thus studying further how epithelial cells keep their own polarity will provide clues to therapeutic methods to prevent carcinogenesis or even make cancer cells back to normal epithelial cells, or cells with a property of benign tumor, at least.

### 5.2. Role of Laminin and Dystroglycan in OLG Polarization, Differentiation, and Myelination

 Laminin-2 deficiency causes peripheral myelination defects in human and mice [301–303]. Also central dysmyelination, delayed OLG differentiation, and increased death of OPCs were reported in laminin-2 deficient (dy/dy) mice [304–306]. As laminin receptors, OLGs express integrin alpha6beta1 and dystroglycan [307, 308]. In the absence of dystroglycan, primary OLGs showed substantial deficits in their ability to differentiate and to produce normal levels of myelin-specific proteins [308]. By blocking the dystroglycan receptors, OLGs failed both to produce myelin-like membrane sheets and to initiate myelinating segments when cocultured with dorsal root ganglion neurons [308]. The effects of dystroglycan may be via IGF-1, because loss of dystroglycan led to a reduction in the ability of IGF-1 to activate MAPK signaling, and dystroglycan interacted with the adaptor protein Grb2 and insulin receptor substrate-1 (IRS-1) [286]. In addition, dystroglycan promotes filopodial formation and process branching in differentiating OLGs [288], suggesting that dystroglycan may play a role in polarization of OLGs during development before myelination process starts. In contrast, antibodies against integrin beta1-subunit inhibit myelination in vitro and in vivo [309, 310]. Enhanced OLG survival in response to the ECM, in conjunction with growth factors, was dependent on interactions with beta1 integrins and did not require dystroglycan [308]. Also, in beta1-integrin transgenic mice studies, beta1-integrin was shown to have a role in the axo-glial interactions regulating myelination [311–313]. These data suggest that integrin and dystroglycan play a differential role in myelination in OLGs as these proteins do in Schwann cells.

Laminin influences several signaling proteins in cultured OLGs, including Fyn, FAK, MAPK, and PI3K [304, 305, 314–317]. These laminin-induced signalings seem to be mediated mostly by integrin. For example, laminin induces Fyn activation, probably via integrin, and then FAK may promote OLG process outgrowth in a Fyn-dependent manner [305, 317]. Recently, localization of dystroglycan in focal adhesions was reported [318]. Also interaction of dystroglycan with FAK is suggested in OLG process [288] as well as in brain synaptosomes [319]. Thus dystroglycan-FAK interaction may play a role in OLG process outgrowth. However, it remains unclear how laminin-induced signaling plays a role in specific aspects of OLG polarization and differentiation, including process formation and branching, localization of polarity proteins, or myelin protein trafficking. One interesting candidate is laminin signaling via Fyn or QKI. Relucio et al. reported that laminin contact with OPCs suppresses p27 accumulation and p27 is increased in laminin-deficient brains [306]. Integrin has been shown to regulate p27 by activating signaling which controls p27 protein degradation [320]. The RNA binding protein, QKI, functions in MBP mRNA nucleo-cytoplasmic transport as well as mRNA stabilization of MBP and p27 [168, 321]. Fyn is known to phosphorylate QKI and regulate its ability to stabilize and traffic MBP mRNA [322]. In addition, QKI is suggested to regulate PLP expression as well as Sirt2 [173]. Sirt2, atypical HDAC playing multiple roles in cells, is now emerging as one of the major components of the myelin proteome [161, 323, 324]. Sirt2 controls process arborization of differentiating OLGs in vitro [323]. Sirt2 variant2 (v2) is localized in the paranodal and compact myelin in proximity of PLP and DM20 [161, 323, 324]. PLP/DM20 is suggested to regulate SIRT2 transport into myelin [161]. It is worth noting that Sirt2 modulates Schwann cell myelination via regulating PAR3/aPKC signaling [325]. Thus, laminin signaling seems to be mediated at least partly by Fyn, QKI, p27, and Sirt2, and these complex interactions may promote OLG polarization and myelination in multiple ways.

### 5.3. Role of Dystroglycan in Schwann Cell Myelination and Polarity

In Schwann cells, alpha- and beta-dystroglycan constitute a membrane-spanning complex, dystrophin-glycoprotein complex (DGC), in abaxonal membrane, and interact with various cytoplasmic and transmembrane proteins and components of basement membrane, in particular, laminin-2 [303]. The way that dystroglycan is expressed in Schwann cells is strikingly different from OLGs. In Schwann cells, dystroglycan appears in abaxonal membrane along with the formation of basement membrane as well as the expression of laminin-2 in perinatal period and continues to be present in abaxonal membrane, interacting with laminin-2, a major component of basement membrane, in mature Schwann cells. In OLGs, there is no classic basement membrane in mature cells, and localization of dystroglycan during development of OLGs remains unclear [326]. Nevertheless, functional roles of laminin-2 and dystroglycan are similar between these two types of myelinating glia. 

There is ample evidence suggesting that dystroglycan plays a role in radial sorting and myelination in Schwann cells through interaction with its ligand, laminin-2. First, expression of dystroglycan and laminin-2 dramatically increases at the period of myelination in both development and nerve regeneration [327, 328]. Second, conditional knockout of dystroglycan in Schwann cells showed not only nodal changes such as reduced sodium channel density and disorganized microvilli but also abnormal myelin sheath folding, suggesting dystroglycan is necessary for myelin maintenance [329]. Third, mice phenotype of Schwann cell-specific ablation of both alpha6beta4 and dystroglycan was compared with that of alpha6beta4 ablation only. As a result, the former showed more evident myelin folding abnormalities as well as acute demyelination, suggesting that alpha6beta4 integrin and dystroglycan cooperate to stabilize myelin sheath [330]. Fourth, absence of dystroglycan in a specific genetic background causes sorting defects similar to those of laminin-2 mutants. Whereas absence of beta1 integrin impaired proliferation and survival and arrests radial sorting at early stages, absence of dystroglycan seemed to arrest radial sorting at a later step [331]. Thus, the functions of these two receptors in radial sorting are not redundant, but sequential.

However, the molecular mechanism of how dystroglycan regulates myelination remains unknown. Because dystroglycan is suggested to play a role in polarization of several types of cells including OLGs as described previously, dystroglycan may regulate myelination via its effects on Schwann cell polarization. However, very little is known about whether signaling induced by laminin-dystroglycan interaction or DGC itself plays a role in Schwann cell polarization, or in polarized localization of polarity complexes. As reported in other types of cells [283–285, 288], dystroglycan may play a role in Schwann cell polarization by regulating small GTPase signaling, such as Rac1 and Cdc42, which also interacts with polarity complexes.

## 6. Translation of the Achievements in Epithelial Cells and OLGs into Polarization/Myelination Process of Schwann Cells 

### 6.1. A Model of Schwann Cell Polarization and Myelination

Myelin sheath is a terminally differentiated structure of Schwann cells closely associated with Schwann cell radial polarity. In order to form myelin sheath, it is required that myelin specific lipids such as sphingolipid, cholesterol, and myelin-associated proteins are specifically transported to abaxonal membrane domain. Actually, GalCer and sulfatide are present on the surface of Schwann cells at least 1 day before myelination starts [332, 333]. It is, therefore, plausible that Schwann cell polarization is a prerequisite for myelination to start, or progresses coordinately with myelination, at least. 

Fundamental machineries for establishment of cellular polarization seem to be highly conserved during evolution. Thus, translation of epithelial cell polarization into the developmental process of Schwann cell will be useful for elucidating the mechanism of Schwann cell polarization/myelination. (The readers interested in Schwann cell developmental process are referred to other reviews [334–337].) First, during development, Schwann cells proliferate, migrate, and ensheath bundles of axons. Once these axons are completely encircled by Schwann cells, basement membrane is formed around each Schwann cell. Then, radial sorting starts, and 1 : 1 relationship between Schwann cell and axon is eventually established. Triggered by signals from axons such as those induced by neuregulin1, polarization of Schwann cells should start. Thus, Schwann cell polarization may start when Schwann cells completed the ensheathment of axon bundles. Adaxonal membrane domain is first determined by contact with axon, and then abaxonal domain is determined, commencing the formation of basement membrane, which is a prerequisite for radial sorting to start [338]. Concomitantly, intrinsic polarity complexes, such as PAR, Crumbs, and Scribble, are recruited to their correct intracellular position. During radial sorting, Schwann cells go through complicated morphological rearrangement. When the polarized localization of the polarity complexes is completed is unknown. It may be completed at the early stage of radial sorting, and the polarity complexes may regulate radial sorting by interaction with beta1-integrin/Rac1 and dystroglycan [134, 139, 330, 331, 339, 340]. Or, at the latest, when the 1 : 1 relationship is established, the polarized localization of these polarity complexes may be completed, and they are ready to provide intrinsic cues advancing the polarization process further, such as sorting and transport of myelin-associated lipid and proteins. When all kinds of myelin components are transported to and accumulated in adaxonal membrane, myelin membrane wrapping will start.

### 6.2. Perspectives in Schwann Cell Polarization and Myelination

Understanding the roles of Schwann cell polarization in myelination is just beginning. Overviewing the findings accumulated so far in the polarization studies for myelinating glia and other cells led the author to emphasize two aspects of Schwann cell polarization. One is the polarization network centered around polarity complexes and small GTPase signaling. The other is the polarization regulated by myelin protein trafficking including transport, exocytosis and endocytic recycling. Each topic will be discussed separately first.

As for the former aspect, polarized localization of three polarity complexes, PAR, Crumbs, and Scribble complexes in Schwann cells, has been revealed. The three polarity complexes are master regulators of cell polarization, and cross-regulation with small GTPase signaling proteins, such as Rac1 and Cdc42, has been suggested. On the other hand, Rac1 and Cdc42 have been suggested to play an important role in radial sorting and myelination in Schwann cells and also are regulated by laminin signaling [341]. Thus, interactions between PAR complex and small GTPase signaling seem to comprise one of the core parts of Schwann cell polarization/myelination protein network. Recently, evidence showing the importance of PAR3 in myelination is accumulating. PAR3 was found to be localized at adaxonal domain (axon-glia junction) at the onset of myelination. There, PAR3-p75NTR interaction seems to play a role in initiation of myelination process via Rac1 [342–344]. In addition, overexpression of PAR3 or PAR6 as well as knockdown of PAR3 inhibits myelination. Sirt2 was identified as an essential regulator of PAR3 activity during myelin formation in Schwann cells [325]. Thus, not only the expression of PAR3, but also localization of PAR3 at the adaxonal domain is necessary for myelination to start. Also PAR3 may regulate myelination by bidirectional interaction with ROCK [345–347]. Extrinsic cues provided by laminin may regulate myelination by modifying small GTPase signaling [341]. Hypothetically, for example, PAR3 recruits PTEN which produces PtdIns(4,5)P2 to adaxonal membrane. Then, PtdIns(4,5)P2 recruits Cdc42 and Par complexes via scaffold proteins such as annexins [150]. The recruited Cdc42 and Par complexes in adaxonal membrane can regulate polarized trafficking including endocytosis and exocytosis. Also it is worth noting that Dlg1-PTEN interaction regulates myelin thickness by inhibiting AKT activation induced by neuregulin1 signaling from axon [348]. Also Dlg1 is suggested to regulate myelin thickness by interacting with Kif13B, Mtmr2 (regulator of endocytosis), and Sec8 (exocyst component) [349]. By those interactions, Dlg1 may regulate balance between Sec8-mediated membrane addition and Mtmr2-mediated negative control of membrane formation. Scribble complex and Par complex colocalize at sites of membrane remodeling in migrating epithelial cells. Thus, Scribble localized at abaxonal domain in matured Schwann cells may be recruited to adaxonal domain during myelination process. Considering their antagonistic role and their interaction with Rac1 [134, 139], a possibility was suggested that dynamic switching of Rac activity may be regulated by the Scribble and Par complexes [147]. Thus, neuregulin signaling from axons mediated by PAR3/Dlg may play a role in regulating myelin thickness in the similar way that Scribble and PAR complexes regulate epithelial migration. Importantly, Dlg1-silenced Schwann cells often failed to myelinate in vitro [348]. These cells also showed migration defects and expressed less PAR3. Altogether, Dlg may play roles in both initiating myelination via interaction with PAR3 and regulating myelin thickness during the process of myelination via interaction with PTEN.

**Figure 1:**
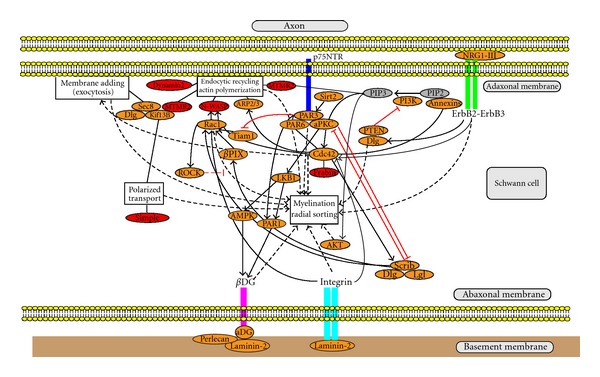
Hypothetical protein network regulating Schwann cell myelination/polarization: complex interactions among Par complex, Dlg, PI3K-signaling-associated components such as PTEN and small GTPase signaling proteins such as Cdc42 and Rac1 seem to play an important role in regulating Schwann cell polarization/myelination. Red ellipse indicates proteins associated with CMT.

Based on the findings accumulated so far, including those discussed previously, hypothetical Schwann cell polarization/myelination network is shown in Figure 1. So far, complex interactions among PAR complex, Dlg, and PI3K-signaling-associated components such as PTEN and small GTPase signaling proteins such as Cdc42 and Rac1 seem to play an important role in regulating polarization/myelination (Figure 1).

In addition, by analogy with epithelial cells, there are many small questions to be answered. Does neuregulin signaling direct the polarized localization of polarity complexes and how? As PAR3 is recruited to tight junctions through PAR3-JAM interaction [126], do the polarity proteins interact with cell junctional proteins in Schwann cells? As mRNA for Crumbs and Pals1 are enriched near the apical surface, do mRNA of polarity proteins show polarized localization in Schwann cells? As in other type of cells, do polarity complexes actively exclude each other? Does laminin or DGC have effects on polarized localization of polarity proteins? When do polarity proteins show polarized localization during development? How does the polarized localization of polarity proteins change in axonal injury inducing Schwann cell dedifferentiation? 

Another important aspect is myelin protein trafficking including transport, exocytosis, and endocytotic recycling. Owing to the studies pursuing the pathomechanisms of CMT, ample evidence accumulated showing how myelin protein is transported to myelin membrane domain. In summary, MPZ is abundantly present in late endosomes/lysosomes, and downregulation of Rab27a, required for the trafficking of the secretory lysosomes to the plasma membrane, blocked lysosomal exocytosis in Schwann cells and at the same time reduced remyelination in regeneration [232]. Several types of Charcot-Marie-Tooth diseases are caused by mutations of genes associated with membrane trafficking system, MTMR2, which regulate endocytosis, DNM2, regulating membrane trafficking from TGN, and SIMPLE, regulating lysosomal sorting and protein degradation. Altogether, these results strongly suggest that myelin protein transport is mediated by membrane trafficking system which is also used in protein transport for cell polarization and that myelin protein transport is critically important for Schwann cell polarization, a prerequisite of myelinogenesis. Especially, mutations of MTMR2 and DNM2 in CMT suggest the importance of endocytic recycling of myelin proteins. Actually, N-WASP, which is a downstream effector of Rac1 and Cdc42 and regulates actin polymerization or endocytosis via Arp2/3, is required in Schwann cell myelination [350, 351] (Figure 1). Further study is necessary to reveal detailed molecular mechanisms of myelin protein sorting, transport, targeting to myelin membrane domain, and endocytotic recycling. Considering the fact that many GPI-anchored proteins including MPZ, MAL, and PMP22 are present in myelin sheath, transport associated with lipid raft may play an important role in myelin protein transport in Schwann cells. Examining detergent solubility of myelin proteins in various Schwann cell developmental stages may help to clarify the role of lipid raft.

 In this context, another important question is how polarity complexes interact to cross-regulate the trafficking machinery. Whereas it remains a mystery in general, Cdc42 is suggested to be the one candidate molecule integrating these two networks [352]. Cdc42 regulates exocytosis in secretory cells [353]. In cyst-forming MDCK cells in 3D culture system, Cdc42 seems to regulate polarized trafficking from TGN [354, 355]. In *Drosophila*, Cdc42 may regulate apical trafficking and endocytosis at adherens junction via interaction with Par complex or Crumbs [115, 116, 118]. Cdc42 along with Cip4 may regulate endocytosis at adherens junction via interaction with WASp, Arp2/3, and dynamin functioning in vesicle scission or “pinching” [115, 118]. Thus studying the functions of Cdc42 associated with vesicle trafficking, exocytosis and endocytosis in Schwann cells will provide important clues to not only the mechanism of myelination but also the pathogenesis of CMT.

By analogy with OLGs, studying functions of QKI in Schwann cells will provide important clues to Schwann cell polarization and myelination. Although quaking viable (qk^*v*^) mutant mice show only mild hypomyelination in PNS in spite of significant reduction of all qkI mRNA isoforms [356], it does not always mean that QKI proteins do not play an important role in PNS myelination. There may be different compensation mechanisms between Schwann cells and OLGs. Studying QKIs will reveal whether MBP is transported as mRNA also in Schwann cells, or whether QKI mediates laminin signaling also in Schwann cells. In addition, a bioinformatic analysis using QRE (QKI response element) identified more than 1400 putative mRNA targets, of which many encode proteins crucial for OLGs and myelin development [170]. Studying functions of these proteins in Schwann cells will reveal differences of myelination machinery between Schwann cells and OLGs.

Finally, it is worth noting that overall mechanism of cell polarization is just beginning to be understood and further research for unknown components of polarity in Schwann cells is necessary. Accordingly, systematic analysis for transcriptome or proteome will be critically important in identifying unknown components belonging to myelin proteome or polarity proteome in Schwann cells. At the same time, analyses dissecting protein interactions between each component will reveal overall Schwann cell-specific polarization/myelination network.

## 7. Conclusion

 Understanding the Schwann cell polarization is just beginning. Also understanding cell polarization process remains incomplete even in the cells most extensively studied such as epithelial cells, and the field of cell polarization remains to be one of the hottest fields in biology. Translating the findings of cell polarization in other cell types into Schwann cell biology may be an efficient strategy for elucidating the mechanism of Schwann cell polarization. So far, accumulating evidence suggests that complex interaction among Par complex, Dlg, and PI3K-signaling-associated components such as PTEN, and small GTPase signaling proteins plays an important role in regulating polarization/myelination in Schwann cells. On the other hand, studies pursuing the mechanism of CMT have suggested that myelin protein trafficking plays an important role in Schwann cell polarization/myelination. For revealing Schwann cell specific molecular network of polarization/myelination, it is useful to combine systemic approach identifying unknown components of polarity/myelin proteome in Schwann cells and analyses dissecting protein interactions between each of the components.
